# Kangaroo Stimulation Game in Tracheostomized Intensive Care–Related Dysphagia: Interventional Feasibility Study

**DOI:** 10.2196/60685

**Published:** 2025-03-05

**Authors:** Marjolein Jansen, Ingrid D van Iperen, Anke Kroner, Raphael Hemler, Esther Dekker-Holverda, Peter E Spronk

**Affiliations:** 1 Expertise Center for Intensive Care Rehabilitation Apeldoorn (ExpIRA), Gelre Hospitals, Apeldoorn, The Netherlands; 2 Department of ICU, Gelre Hospitals, A.Schweitzerlaan 31, Apeldoorn, 7334DZ, The Netherlands, 31 555818450; 3 Department of ENT, Gelre Hospitals, Apeldoorn, The Netherlands; 4 Department of Speech and Language Pathology, Gelre Hospitals, Apeldoorn, The Netherlands

**Keywords:** dysphagia, swallowing, intensive care, ICU, swallowing disturbance, kangaroo stimulation, game, feasibility study, surface electromyography, training, exercise, Rephagia biofeedback, muscle strength, stamina, timing, tracheostomy, clinical, feasibility

## Abstract

**Background:**

Dysphagia is common in intensive care unit (ICU) patients. Using surface electromyography (sEMG) signals as biofeedback training exercises might offer a promising path to improving swallowing function. The Rephagia biofeedback system uses sEMG to assess muscle strength, stamina, and timing of the swallowing action.

**Objectives:**

The aim of this study was to evaluate the feasibility of the Rephagia system in ICU patients with dysphagia.

**Methods:**

This feasibility study included patients admitted to a 14-bed mixed medical-surgical ICU. All patients underwent a new tracheostomy placement during ICU stay due to persistent aspiration and ICU-acquired weakness, accompanied by verified dysphagia. Following Rephagia training, patients completed a questionnaire assessing comprehension, satisfaction, and motivation. Swallowing characteristics were assessed via mean sEMG peak values during exercise.

**Results:**

Twenty patients with a mean age of 69.4 (SD 8.2) years were included. The means of sEMG values at the beginning of a measurement were not significantly different at baseline versus everyone’s last measurement (52 µV [23 µV] vs 57 µV [22 µV]; *P*=.50). The means of sEMG values obtained at the end of a measurement were not significantly different at baseline versus everyone’s last measurement (56 µV [18 µV] vs 59 µV [23 µV]; *P*=.62). However, dysphagia improved in all patients. Patients understood the importance of the game in relation to their swallowing problems (16/80, 89%), which kept them motivated to participate in the training sessions (9/18, 50%).

**Conclusions:**

The Rephagia biofeedback system for stimulating swallowing actions in tracheotomized ICU patients with dysphagia is feasible. No relation was found between clinical improvement in swallowing function and sEMG signals.

## Introduction

Swallowing disturbances occur in up to 30% of hospitalized patients with a length of stay longer than 48 hours [1]. Swallowing in general and the oral, pharyngeal, and esophageal phases of this process require a complex interaction between the neuromuscular system in the tongue, floor of mouth, pharynx, and larynx [2]. This intricate physiological course of muscle events and interactions may become disturbed in patients treated in the intensive care unit (ICU). Swallowing impairment or dysphagia is not uncommon in ICU patients with a reported prevalence of up to 62% of those who were recently extubated [3]. Dysphagia in ICU patients is probably caused in part by muscle weakness, sensory neuropathy, and cognitive disturbance due to critical illness but possibly also in part by inadvertent trauma to laryngeal structures due to endotracheal intubation [4]. Dysphagia is associated with prolonged length of hospital stay and the composite end point of increased risk of pneumonia, reintubation, or death. This significantly affects the patient’s well-being, ICU resources, and health care costs [5]. It has also been hypothesized that certain interventions used to prevent aspiration, such as cuffed tracheostomies, might in the long run actually maintain or worsen dysphagia due to disturbance of normal laryngeal anatomy and sensibility [6].

The prevalence of dysphagia is frequently underestimated and interventions to improve swallowing function are not evident [7,8]. Most frequently used interventions include exercises such as repetitive swallowing, chin tuck against resistance, and respiratory exercises [9]. Recently, electrical stimulation of the pharyngeal muscle groups has been shown in one center to be promising, especially in neurological patients [10]. Muscle activity can be observed as surface electromyography (sEMG), although its interpretation is complex [11]. In parallel, it is intriguing to consider the use of sEMG signals as biofeedback training exercises to affect different parts of the swallowing process including laryngeal lifting, oral transit time, and excursion of the hyoid bone. Indeed, this approach has been shown to be feasible in patients with several diseases including Parkinson disease and those who experienced a stroke [12-14]. The Rephagia biofeedback system uses sEMG to assess muscle strength, stamina, and timing of the swallowing action and visualizes this using a graph. Furthermore, the sEMG signal from the patient may be used in an interactive game using various exercises in which timing and strength of the swallowing action are trained.

We hypothesized that the Rephagia biofeedback system using an interactive game to evaluate and stimulate swallowing is feasible and potentially useful to evaluate dysphagia over time in ICU patients.

## Methods

### Setting

This study was designed as an uncontrolled prospective effectiveness and feasibility study between August 2017 and September 2021 and was undertaken in the 14-bed mixed medical-surgical ICU of Gelre Hospitals Apeldoorn, a nonacademic university–affiliated teaching hospital. The team of ICU care professionals including the speech and language professionals and ear-nose-throat consultants did not change during the study.

### Patients

Patients were included if they had a new tracheostomy (Tracoe cannula with cuff) placed during ICU stay due to persistent aspiration and ICU-acquired weakness and did not have a history of swallowing impairment or other dysphagia symptoms. Patients were considered for inclusion as soon as they were in the process of weaning from the ventilator and could be detached from the ventilator for at least 2 consecutive hours. Patients were excluded if language barriers existed, or if permanent cognitive impairment was present. In addition, patients with severe visual impairment were excluded.

### Ethical Considerations

The study was approved by the institutional ethical review board (no. TCO 16.10) in accordance with institutional and national guidelines for research involving human participants. Ethical approval covered all aspects of the study, including patient recruitment, data collection, and analysis. Eligible patients or, if applicable, their legal representatives were verbally informed about the study and given the opportunity to ask questions. After being invited to participate, the patients and their legal representatives received an information pamphlet detailing the study procedures. They were given at least 30 minutes to consider participation before signing an informed consent form. Patients were allowed to cease participating at any time if they did not want to continue. All patient data were anonymized to ensure confidentiality. Identifiable information was not included in the study records or publications. Patients were observed closely while assessing swallowing and the test was paused or discontinued if swallowing safety was compromised or at the request of the participant. No financial or other compensation was provided to the participants.

### Procedure

If there was any suspicion of dysphagia, the speech and language pathologist (SLP) was contacted and initiated the flexible endoscopic evaluation of swallowing (FEES), which was performed by the ear, nose, and throat specialist together with the SLP. All patients underwent the standardized FEES examination using the Murray Secretion Scale [15] and the Rosenbek Penetration Aspiration Scale (PAS) [16] on thin liquid, thick liquid, and regular food if safe. For thin liquid, water with blue dye was used and for thick liquid, regular yoghurt was used.

Dysphagia was judged to be present if Murray Secretion Scale score was 2 or higher and PAS score was higher than 2 in thin, modified liquid or regular food [17]. If FEES demonstrated dysphagia or aspiration, patients were eligible for enrollment in the next step of the study. After the FEES, Rephagia training was started as soon as possible. All participating patients were asked after the first Rephagia training to fill out a questionnaire with questions concerning understanding, satisfaction, and motivation using a modified tool (Table S1 in Multimedia Appendix 1).

**Figure 1.**
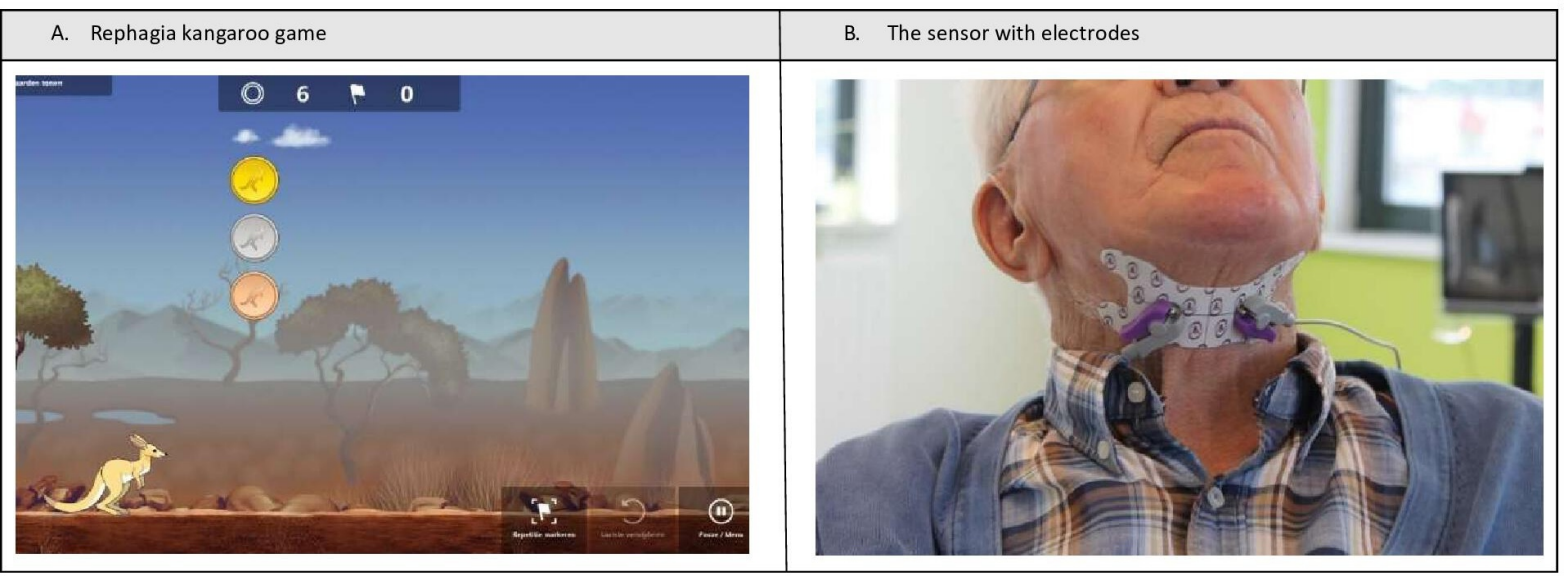
The Rephagia kangaroo game (A) and the sensor with electrodes (B).

Subsequently, daily training and exercise was performed using the Rephagia kangaroo game (Figure 1A). Settings (bolus size, bolus consistency, and how many swallowing actions) of the exercise were defined by an experienced SLP, using a specified protocol as described previously [1]. The daily Rephagia swallowing exercises were supervised by a trained ICU nurse. In addition, all patients were evaluated once weekly by an SLP. Parameters related to acquired sEMG in relation to clinical parameters such as swallow timing and strength were stored for later analysis. The daily training session was paused if one of the following safety impairments occurred: coughing, wet voice, and decrease of oxygen saturation of 3% or more on the finger pulse oximeter. The training sessions were terminated if improvement of swallowing had not occurred after 3 weeks of training sessions, or if the patient had been successfully decannulated.

### Swallowing Characteristics and Rephagia System

Besides the initial FEES analysis, swallowing characteristics were assessed using the sEMG data provided by the Rephagia biofeedback (SilverFit). These data may be used as a tool to characterize swallowing action using the sEMG peaks in individual patients. It is a noninvasive but sensitive method to identify a swallowing action. An electrode with a sensor for the sEMG signals was attached to the submandibular region to assess the swallowing characteristics (Figure 1B). The sensor was connected to a laptop with the Rephagia software via Bluetooth. The signals from the sensor were used in an interactive game. Before the start of the actual game, the sEMG signals were calibrated for each individual patient and each test by obtaining 3 normal swallows to obtain an individual baseline value. In the game, the patient received feedback of the swallow activity in the form of a hopping kangaroo. The administered test consisted of repetitions where the patient was asked to swallow when the kangaroo stopped at a coin and waited for the patient to swallow.

The swallows were repeated 10‐25 times on individual basis using bolus volume and type of food prescribed by an experienced SLP. In this study, swallow characteristics were obtained using the mean peak values of swallow actions of the participants obtained over the course of an exercise. The first 3 swallow actions and the last 3 swallow actions were used to correct for individual variation on the one hand and to gain insight in individual swallowing characteristics and changes in the individual swallowing characteristics during the exercise on the other hand. The first 3 swallow actions are described as the beginning of a measurement and the last 3 swallow actions are described as the end of a measurement. Normal values of sEMG were previously described in healthy individuals as mean 82 μV (SD 33 μV) [18].

### Statistical Analysis

Descriptive statistics were generated for all outcome measures. Data distribution was tested for normality using the Shapiro-Wilk test. Accordingly, variables were described as mean and SD or as median and IQR. Categorical variables were described using frequencies and percentages. The analysis of the comparison of baseline swallowing characteristics with the swallowing characteristics of the last swallowing measurement was conducted using the paired 2-tailed *t* test or the Wilcoxon signed rank test depending on the normality of data distribution. The change in swallowing characteristic within a measurement at baseline versus the last measurement was also analyzed using either the paired 2-tailed *t* test or the Wilcoxon signed rank test.

Individual changes in swallowing characteristics were analyzed by calculating the percentage of measurements higher or lower than the baseline measurements. Data were analyzed with the statistical package statistical package SPSS (version 25; IBM Corp), Excel (version 2202; Microsoft Corp), and R (version 4.0.4; R Foundation for Statistical Computing). A *P* value of <.05 was considered significant.

## Results

### Patients

After screening 58 patients, 20 patients were formally included. Thirty-eight patients could not be included because of the following reasons: no informed consent (N=4); too late for inclusion (N=4); no FEES possible due to the lack of trained ear, nose, and throat specialist or SLP present (N=4); preexistent swallowing problems; cognitive, neurological, or vision problems; or delirium (N=11). Fifteen patients were subsequently excluded from study inclusion after the FEES because no signs of dysphagia were found. All outcome parameters were recorded prior to participation at baseline until end of the study (Table 1). Mean age at baseline of the included patients was 69.4 (SD 8.2) years. The mean ICU length of stay was 52.0 (SD 21.9) days. In 12 patients, the PAS score for their ability to swallow thin liquid was assessed. All 12 patients demonstrated scores higher than 2 on the PAS, classifying as dysphagia. In 18 patients, the PAS score for their ability to swallow thick liquid was assessed. Seventeen patients obtained scores higher than 2 and 1 patient obtained a score of 1. The Murray Secretion Scale score was assessed in 17 patients. Two patients obtained a normal rating, 3 patients had secretions outside the laryngeal vestibule that were cleared with spontaneous swallows, 4 patients had deeply pooled secretions, and 8 patients had secretions in the laryngeal vestibule that were not cleared. The comprehensive PAS scores and Murray Secretion Scale scores are shown in Table S1 in Multimedia Appendix 2. One month after the end of the study, patient charts were reviewed. There was no occurrence of pneumonia, ICU readmission, and reintubation. All patients were successfully decannulated. Patients were decannulated based on clinical observations made by registered nurses and assessments by SLPs. Two patients died during the ICU admission, not related to swallowing abnormalities.

**Table 1.** Patient characteristics^a^.

Characteristics		Values
**Sex, n (%)**		
	Male	13 (65)
	Female	7 (35)
Age (years), mean (SD)		69.4 (8.2)
**Reason for admission, n (%)**		
	Medical	16 (80)
	Emergency surgery	3 (15)
	Elective surgery	1 (5)
Hospital length of stay (days), mean (SD)		74.3 (23.0)
ICU^b^ length of stay (days), mean (SD)		52.0 (21.9)
Number of practice moments, median (IQR)		4.0 (3.0-6.2)
**Confirmed infection during hospital stay, n (%)**		
	No	2 (10)
	Yes	18 (90)
SAPS II ^c^ score, mean (SD)		44.2 (14.7)
**Mechanical ventilation at admission^d^** **, n (%)**		
	No	13 (65)
	Yes	7 (35)
**Mechanical ventilation within 24 hours, n (%)**		
	No	4 (20)
	Yes	16 (80)
Hours on mechanical ventilator, median (IQR)		978.5 (638.7-1257.8)
Days witch tracheostomy^e^, median (IQR)		31.0 (22.4-95.1)
APACHE II score, mean (SD)		19.9 (7.2)
APACHE III score, mean (SD)		76.1 (28.8)
Predicted mortality (%)^f^, mean (SD)		34.1 (22.5)
**Died, n (%)**		
	No	18 (90)

a Variables with a normal distribution are described as mean (SD) and variables with a nonnormal distribution are described as median (IQR). For the categorical variable, count (n) and percentage (%) are presented.

b ICU: intensive care unit.

c SAPSII: Simplified Acute Physiology Score.

d Use of a ventilator at the moment of intensive care admission or immediately (within 15 minutes) thereafter.

e Number of days from the placement of the tracheostomy until decannulation.

f Predicted mortality score according to the APACHE IV prediction model (in percentages).

### Swallow Characteristics

No safety-related events occurred during the training sessions. The total duration of the exercise with Rephagia training was 20 minutes per session. The median number of practice sessions was 4 [3-6] per patient. Details for the participating patients are shown in Table 1. The means of sEMG values at the beginning of a measurement were not significantly different at baseline versus everyone’s last measurement (52 µV [23 µV] vs 57 µV [22 µV]; *P*=.50). Similarly, the means of sEMG values obtained at the end of a measurement were not significantly different at baseline versus everyone’s last measurement (56 µV [18 µV] vs 59 µV [23 µV]; *P*=.62).

The means of the sEMG values at the beginning of a measurement were not significantly different from the means of the sEMG values at the end of a measurement when looking at baseline compared with everyone’s last measurement (3.7 µV vs 2.6 µV; *P*=.72).

In 2 patients, the PAS score was assessed during a follow-up FEES after decannulation. The PAS scores of the first patient were 8 on both thin and thick liquid and 1 after decannulation on both consistencies. The PAS scores of the second patient were 7 on thin liquid, 3 on thick liquid, and 1 after decannulation on both consistencies.

**Figure 2.**
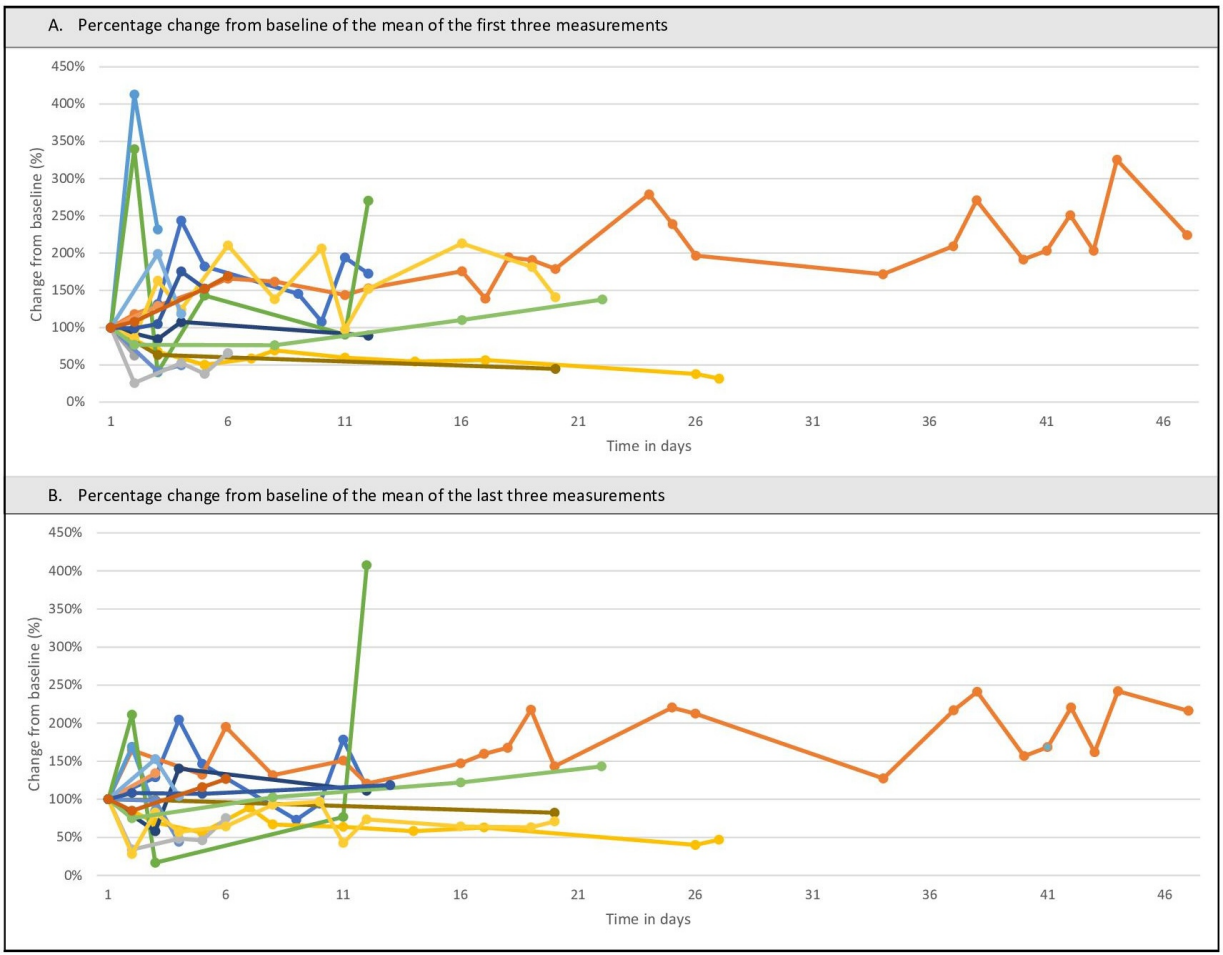
The percentage change from baseline of the surface electromyography data of the mean swallows of the (A) first 3 measurements and the (B) last 3 measurements.

In Figure 2 all individual sEMG signals are depicted. The means of the swallows during each practice moment are shown as a percentual change from the patients’ own baseline, which are plotted against the time in days. No clear trend can be seen in these graphs.

### Patient Satisfaction and Motivation

**Figure 3.**
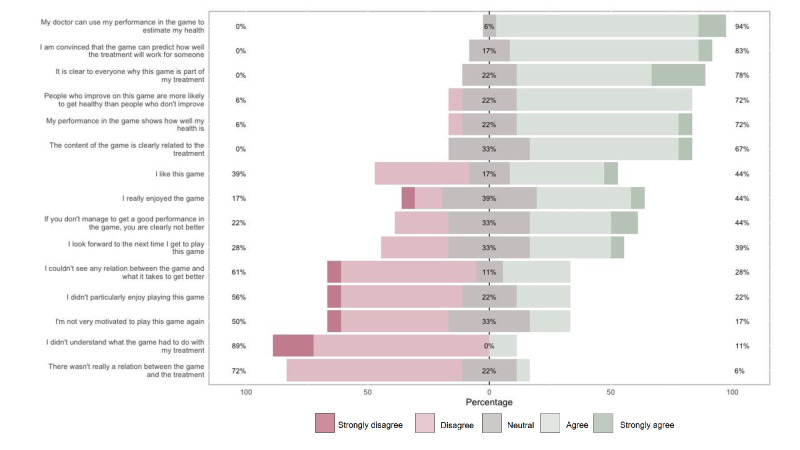
Summary response of the patient questionnaire.

Our findings demonstrate that patients recognize how important swallowing training is during ICU stay. The patients understood the importance of the game in relation to their swallowing problems (16/18, 89%), which kept them motivated to participate in the training sessions (9/18, 50%). The findings demonstrate that patients think that the ICU doctor can use the performance in the game to estimate the severity of illness (19/18, 95%) and predict how the treatment will affect their dysphagia (15/18, 83%). The patients were also convinced that the game can predict the effect of the training and in which way this reflects the patients’ health status. The results of the Likert scale questions are summarized in Figure 3.

## Discussion

### Principal Findings

In this study, we showed that the Rephagia biofeedback system using an interactive game for the stimulation of swallowing in dysphagic ICU patients is feasible in an ICU environment. Individual changes in obtained sEMG signals during swallowing actions were not useful to evaluate improvements in dysphagia.

Playing interactive games and engaging patients in an ICU setting are feasible [19,20]. However, the use of a surface electrode using detected EMG signals while performing muscle tasks in the ICU represents a novel approach in this field. Previous studies in healthy participants demonstrated a relation between obtained sEMG signals from the masseter muscle and effortful swallow maneuvers [21]. Moreover, using sEMG signals from masseter and hyoid muscles, age could be identified as a risk factor for dysphagia [22]. Decreased sEMG signals were found to be associated with swallowing disorders in older adult patients with sarcopenia [23]. However, it may also prove difficult to use sEMG signals as derivatives of swallowing function, since different swallow-specific tasks in healthy adults may cause increased or decreased submental muscle activities [24].

All participating patients showed significant improvements in their swallowing function over the course of the study. However, it was disappointing that these clinical improvements did not coincide with observable changes in surface EMG signals during swallows. In previous studies, the benefits of training with biofeedback sEMG were tested over multiple sessions in patients diagnosed with dysphagia [1], whereas a relatively low number of practice sessions was attainable in this study caused by a delayed availability of FEES; no availability of trained ICU nurses; fatigue of patients due to, for example, dialysis, fever, or sickness; other procedures such as computed tomography or magnetic resonance imaging; and family visits. These factors require attention in future studies with the Rephagia system.

Participants did not display a significant increase in surface EMG intensity if the first and last swallow actions were evaluated. However, all patients did clinically improve in terms of swallowing function, resulting eventually in decannulation in all cases. This confirms previous observations in patients with clinical dysphagia compared with those with no dysphagia where no differences in sEMG signals were found between both groups [1].

These results are not so surprising because the sEMG signal is a derivative of isotonic muscle contractions and is characterized by a nonlinear relationship between force and EMG signal. In addition, the coordination and duration of contractions of muscle groups during dynamic muscle contractions are complex [11]. Moreover, other factors are in play as well including improved sensibility and coordination, a compensatory mechanism consisting of smaller sips or bites to swallow or changing the consistency, or a combination of those factors [6].

In the ICU environment, patients may also experience additional muscle atrophy and weakness, which could impact their ability to swallow effectively. Notably, the obtained sEMG signals in this study (around 55 μV) were considerably lower than values obtained in non-ICU patients with swallowing problems (around 105 μV) [1]. However, raw differences in EMG amplitudes across different configurations and electrode placements may not be a valid means to verify muscle activity or intensity, and this could be a contributing factor to the observed differences with non-ICU patients. While older age may also be associated with lower sEMG levels [25], the mean age in both studies was comparable, suggesting that age alone is unlikely to explain the present results. Despite this, clinical improvements in swallowing function can still occur due to adaptive and compensatory strategies, which might not be fully captured by sEMG alone. These voluntary compensatory strategies might involve adjustments in head and neck posture or the recruitment of postural muscles to aid in swallowing. Other approaches using accelerometers as a sensor for biofeedback games may be a useful alternative as was demonstrated in a small study describing the positive effects on swallowing in poststroke dysphagia using a “frog swallows mosquito game” [26]. Moreover, involuntary adaptive movements and displacements in adjacent or related structures may also compensate for swallowing deficiencies in various anatomical areas [27]. In addition to compensatory strategies, improved sensitivity and coordination can contribute to clinical improvements in swallowing. Patients may adopt smaller sips or bites to swallow or modify the consistency of food, and a combination of these factors might lead to enhanced swallowing function [6]. Indeed, all participants in this study learned to modulate their swallowing musculature in response to the biofeedback to play the game. In addition, despite the severe swallowing disorder of the impaired participant, the patients were able to play the game and understood the importance of the game in relation to their swallowing problems which kept them motivated to participate in the training sessions. This confirms our previous experience [28] and is important to engage patients in their personal recovery and rehabilitation activities [29].

### Strengths and Limitations

This study has several strengths. First, it is the first study concerning the feasibility of the Rephagia system in tracheotomized ICU patients in an ICU setting. Second, swallowing characteristics were evaluated in a relatively new manner, using sEMG values by comparing swallowing actions of patients with each other as well as the change in swallow actions in 1 exercise within a group of patients. Third, we quantified the interest of patients in swallowing training, which had not been done before. The positive feedback from participants, together with the encouraging physiological effects, strengthens the evidence of the feasibility of this swallowing training program.

This study has several limitations that should be mentioned. First, it was a small case series performed in 1 center in the Netherlands, limiting the generalizability of the results. Second, we did not track participant adherence with other exercises (or swallowing of food or liquid) given by an SLP or an ICU nurse and therefore do not know how many repetitions of exercises each participant completed. Third, the interventions applied in this study did not specifically target each individual’s underlying deficits in their swallowing physiology, rather the intervention protocol was a predetermined set of swallowing exercise that is adopted in clinical practice. Fourth, the specific EMG parameters corresponding to swallowing time and intensity were not recorded or analyzed in detail. Instead, only the mean EMG values before and after the patient’s training were considered. This approach may not fully capture the nuanced changes in swallowing mechanics and muscle activation over time. As a result, it is difficult to definitively conclude that the observed EMG changes were directly related to improvements in dysphagia, as other factors influencing muscle activity during swallowing may have contributed to the results. Fifth, the questionnaire was administered after the first session of sEMG biofeedback, meaning participants had limited experience with the swallowing exercise. However, this approach was chosen to maximize the number of responses , despite the potential risk of response bias, as participants might have felt inclined to provide more positive feedback to please the researcher. To mitigate this, they were informed that the purpose of the study was to evaluate a new swallowing training, in which the researcher had no vested interest, to determine its potential recommendation for other ICU patients and clinicians. They were also reassured that the questionnaire forms remained anonymous and were analyzed at a later date. Therefore, we think that the risk of response bias was minimized as far as possible.

### Conclusions

The Rephagia biofeedback system for stimulating swallowing actions in dysphagic tracheotomized ICU patients is feasible. Patients understood the importance of the game in relation to their swallowing problems and understood the relationship with their treatment. No relation was found between clinical improvement in swallowing function and sEMG signals obtained by the system. A prospective intervention study is required to assess the potential additional role of the Rephagia system in swallowing rehabilitation in the ICU.

## Supplementary material

10.2196/60685 Multimedia Appendix 1 Patient questionnaires

10.2196/60685 Multimedia Appendix 2 Baseline Penetration Aspiration Scale scores and Murray Secretion Scale

## Abbreviations

FEES: flexible endoscopic evaluation of swallowing
ICU: intensive care unit
PAS: Penetration Aspiration Scale
sEMG: surface electromyography
SLP: speech and language pathologist
