# Supplementary material for: Kangaroo Stimulation Game in Tracheostomized Intensive Care–Related Dysphagia: Interventional Feasibility Study
Source: JMIR Serious Games. 2025 Mar 5;13:e60685. doi: 10.2196/60685 (PMC11902881; doi:10.2196/60685)
Supplement: Multimedia Appendix 1 [file games-v13-e60685-s001.docx]

Multimedia Appendix 1

Table S1. Patient questionnaire.

|  | Strongly disagree | Disagree | Neutral | Agree | Strongly agree |
| --- | --- | --- | --- | --- | --- |
| I did not understand what the game had to do with my treatment |  |  |  |  |  |
| If you do not manage to get a good performance in the game, you are clearly not better |  |  |  |  |  |
| I really enjoyed the game |  |  |  |  |  |
| I could not see any relation between the game and what it takes to get better |  |  |  |  |  |
| I am convinced that the game can predict how well the treatment will work for someone |  |  |  |  |  |
| I am not very motivated to play this game again |  |  |  |  |  |
| It is clear to everyone why this game is part of my treatment |  |  |  |  |  |
| My performance in the game shows how well my health is |  |  |  |  |  |
| I like this game |  |  |  |  |  |
| The content of the game is clearly related to the treatment |  |  |  |  |  |
| I did not particularly enjoy playing this game |  |  |  |  |  |
| There was not really a relation between the game and the treatment |  |  |  |  |  |
| My doctor can use my performance in the game to estimate my health |  |  |  |  |  |
| I look forward to the next time I get to play this game |  |  |  |  |  |
