# Supplementary material for: Kangaroo Stimulation Game in Tracheostomized Intensive Care–Related Dysphagia: Interventional Feasibility Study
Source: JMIR Serious Games. 2025 Mar 5;13:e60685. doi: 10.2196/60685 (PMC11902881; doi:10.2196/60685)
Supplement: Multimedia Appendix 2 [file games-v13-e60685-s002.docx]

Multimedia Appendix 2

Table S2. Baseline Penetration Aspiration Scale scores and Murray secretion scale.

| **PAS score** |  | Thin liquid | Thick liquid |
| --- | --- | --- | --- |
|  | 1 Material does not enter the airway | - | 1 (5.6) |
|  | 2 Material enters the airway, remains above the vocal folds, and is ejected from the airway | - | - |
|  | 3 Material enters the airway, remains above the vocal folds, and is not ejected from the airway | - | 3 (16.7) |
|  | 4 Material enters the airway, contacts the vocal folds, and is ejected from the airway | 2 (16.7) | - |
|  | 5 Material enters the airway, contacts the vocal folds, and is not ejected from the airway | 1 ( 8.3) | 4 (22.2) |
|  | 6 Material enters the airway, passes below the vocal folds, and is ejected into the larynx or out of the airway | 1 ( 8.3) | 3 (16.7) |
|  | 7 Material enters the airway, passes below the vocal folds, and is not ejected from the trachea despite effort | 4 (33.3) | 3 (16.7) |
|  | 8 Material enters the airway, passes below the vocal folds, and no effort is made to eject | 4 (33.3) | 4 (22.2) |
| **Murray secretion scale** | 0 Normal rating | 2 (11.8) |  |
|  | 1 Secretions outside the laryngeal vestibule that are cleared with spontaneous swallows | 3 (17.6) |  |
|  | 2 Deeply pooled secretions or any transition between 1 and 3 | 4 (23.5) |  |
|  | 3 Secretions in the laryngeal vestibule that are not cleared | 8 (47.1) |  |
